# Surgical Decision-Making in Cubital Tunnel Syndrome Stratified by Electrodiagnostic Severity: A Reproducible Markov Model With Probabilistic Sensitivity Analysis

**DOI:** 10.7759/cureus.111683

**Published:** 2026-06-28

**Authors:** Michael J Franco

**Affiliations:** 1 Plastic and Reconstructive Surgery, Cooper University Hospital, Camden, USA

**Keywords:** anterior transposition, cost-utility analysis, cubital tunnel syndrome, electrodiagnostic severity, markov model, medial antebrachial cutaneous nerve, probabilistic sensitivity analysis, qaly, simple decompression, ulnar neuropathy

## Abstract

Background

Cubital tunnel syndrome is the second most common compressive neuropathy of the upper extremity. Existing decision analyses generally use simplified decision-tree frameworks. They do not stratify by electrodiagnostic severity and do not model medial antebrachial cutaneous (MABC) nerve morbidity as a durable health state. We developed a reproducible Markov model comparing simple decompression (SD) and transmuscular anterior transposition (TMT), stratified by demyelinating versus axonal disease.

Methods

A five-cycle annual Markov cohort model was constructed from the societal perspective with 3% annual discounting. Patients entered the model as mild/moderate demyelinating or severe/axonal cubital tunnel syndrome. MABC injury was modeled as a permanent state with an annual utility decrement. Probabilistic sensitivity analysis (PSA) used 10,000 Monte Carlo iterations, beta distributions for probabilities and utilities, and gamma distributions for costs. A complete Python implementation is provided as supplementary material.

Results

In the reproducible implementation, SD remained the preferred strategy in mild/demyelinating disease, with higher mean five-year quality-adjusted life year (QALYs) and lower mean cost than TMT. In axonal disease, TMT produced a small mean QALY advantage but remained close to equipoise, with overlapping credible intervals and a higher mean cost. The MABC risk differential narrowed in axonal disease because the SD pathway has greater downstream revision exposure. These findings support SD for demyelinating disease without subluxation and support individualized discussion of TMT in axonal disease rather than a categorical claim of superiority.

Conclusions

This model supports a stratified approach based on electrodiagnostic severity while emphasizing the uncertainty in axonal disease. The strongest conclusion is not that TMT is universally superior in axonal cubital tunnel syndrome, but that the tradeoff changes when revision risk and MABC morbidity are modeled over time. The model is hypothesis-generating and should be validated with prospective electromyography (EMG)-stratified outcome data.

## Introduction

Cubital tunnel syndrome (CuTS) produces a clinical spectrum ranging from intermittent paresthesias to fixed motor weakness, intrinsic atrophy, and clawing [1]. Surgical options include simple in situ decompression, medial epicondylectomy, and anterior transposition [2]. Randomized trials and meta-analyses have generally not demonstrated clear superiority of transposition over decompression in the broad CuTS population, so simple decompression is often favored because it is less invasive and less costly [3,4].

That broad conclusion may not apply equally to all electrodiagnostic subgroups. Demyelinating neuropathy can recover by remyelination, whereas axonal neuropathy requires regeneration and is more vulnerable to delay, recurrence, and reoperation [1]. A decision model that treats all CuTS as one disease may therefore obscure clinically meaningful heterogeneity.

The medial antebrachial cutaneous (MABC) nerve is another under-modeled variable. It crosses the operative field and may be injured during primary or revision cubital tunnel surgery [5-7]. In a revision setting, painful MABC neuroma can be a dominant source of morbidity and can complicate interpretation of persistent or recurrent symptoms [8]. For that reason, the present model treats MABC injury as a durable health state rather than as a transient perioperative complication.

In this analysis, we attempt to present a transparent, reproducible Markov model comparing simple decompression (SD) and transmuscular anterior transposition (TMT) across demyelinating and axonal disease subgroups, with explicit code-based methods that can be inspected, rerun, and modified. Prior decision analyses have addressed cubital tunnel surgery but generally used simplified decision-tree frameworks without electrodiagnostic stratification or durable modeling of MABC morbidity [9,10].

## Materials and methods

Model structure

We constructed a cohort Markov model with five annual cycles and a 3% annual discount rate applied to costs and quality-adjusted life years (QALYs) [11,12]. The model compared SD and TMT in two disease-severity strata: mild/moderate demyelinating disease and severe/axonal disease. Ulnar nerve subluxation was considered a pre-model triage criterion favoring transposition and was not treated as a probabilistic subgroup within the SD arm.

The model states were symptomatic CuTS at entry, postoperative improvement, postoperative good outcome, postoperative partial recovery/plateau, failed or recurrent symptoms undergoing revision, post-revision good outcome, post-revision partial/poor outcome, and MABC injury as a permanent state. Primary MABC injury could occur during the index procedure. Secondary MABC injury could occur through the revision pathway. Patients with MABC injury remained in that state, with an annual utility decrement applied over subsequent cycles.

Model implementation and reproducibility

The model was implemented in Python using NumPy and pandas. The probabilistic sensitivity analysis (PSA) uses 10,000 iterations and random seed 42. Probabilities and utility values are sampled from beta distributions approximated from manuscript point estimates and plausible 95% ranges. Costs are sampled from gamma distributions. Cumulative revision probabilities were converted to annual probabilities using: p_annual = 1 - (1 - p_cumulative)^(1/4), applied across cycles two through five.

The complete Python file supplied with this revision writes four outputs: raw PSA iterations, summary outputs by arm and severity, incremental TMT-versus-SD outputs, and cost-effectiveness acceptability curve (CEAC) points across willingness-to-pay thresholds from $0 to $200,000/QALY.

Model inputs

Table 1 summarizes the model health states and assigned utility ranges.

**Table 1:** Health-state utility inputs used in the reproducible model CuTS: cubital tunnel syndrome.

Health state	Arm	Utility value/range	Basis
Symptomatic CuTS - mild/moderate	Both	0.72-0.74	Song et al. [5]; Teunissen et al. [13]; demyelinating entry state
Symptomatic CuTS - severe/axonal	Both	0.62-0.65	Song et al. [5]; lower bound plus motor deficit/atrophy decrement
Postoperative improving	Both	0.68-0.76 in code	Early recovery state; intentionally conservative to avoid overestimating first-year QALYs
Postoperative good outcome	Both	0.88-0.91	Brauer and Graham [4]; normalized to generic utility scale
Postoperative partial recovery/plateau	Both	0.75-0.78	Residual hand dysfunction analog
Failed primary/recurrent symptoms	Both	0.65-0.68	Persistent postoperative symptoms
MABC injury permanent state	Both	0.68-0.72 with annual decrement 0.05-0.08	Chronic neuropathic pain/painful neuroma analog
Post-revision good outcome	Both	0.82-0.85	Revision success state
Post-revision partial/poor outcome	Both	0.68-0.72	Poor surgical outcome analog

These values represent the health-related quality-of-life weights applied to each Markov state during each annual cycle. The values were derived from published CuTS decision-analysis literature, time trade-off data, hand-surgery utility literature, and modelled estimates where direct cubital tunnel-specific data were unavailable.

Table 2 summarizes the transition probabilities used in the model, including primary MABC injury risk, revision risk, complication rates, and probabilities of good outcome after primary or revision surgery.

**Table 2:** Transition probabilities by surgical arm MABC: medial antebrachial cutaneous.

Parameter	SD	TMT	Source/basis
MABC injury - primary surgery	1-2%	5-8%	Lowe et al. [7]; Benedikt et al. [6]; Mackinnon/Novak [9] context
Overall complication rate - primary surgery	3.60%	9.60%	Wade et al. [14] network meta-analysis
Revision - mild disease, 5-year cumulative	~8%	~4%	Modeled from long-term revision literature
Revision - axonal disease, cumulative >=48 months	11.90%	3.20%	Reichenbach et al. [10] meta-regression
Good outcome after primary surgery - mild disease	83-85%	81-83%	Song et al. [5]; Brauer and Graham [4]
Good outcome after primary surgery - axonal disease	~60%	~70%	Modeled assumption; key sensitivity parameter
Good outcome after revision surgery	~67%	~67%	Revision series estimate
MABC neuroma found at revision	73%	73%	Mackinnon and Novak [9] revision series

These values determine how patients move between Markov states over the five-year time horizon.

Table 3 summarizes the cost inputs used in the model from the societal perspective.

**Table 3:** Cost inputs in 2024 USD, societal perspective USD: United States dollars; OPPS: outpatient prospective payment system; CPT: current procedural terminology; MABC: medial antebrachial cutaneous; OT: occupational therapy; QALY: quality-adjusted life year.

Cost parameter	Value	Basis
Simple decompression operative cost	$2,800-$4,200	CMS OPPS/professional fee estimate for CPT 64718
Transmuscular anterior transposition operative cost	$4,500-$6,800	CMS OPPS/professional fee estimate for CPT 64719 plus longer OR time
Revision cubital tunnel surgery	$6,200-$9,500	Revision neurolysis/transposition complexity estimate
Postoperative rehabilitation	$800-$1,400	Standard OT course estimate
MABC neuroma management	$1,800-$4,200	Injection series and potential operative excision estimate
Indirect productivity cost - mild disease	$1,200-$3,200	Conservative first-year productivity/recovery estimate used in code
Indirect productivity cost - axonal disease	$3,200-$7,600	Return-to-work literature and societal perspective estimate

These include primary operative costs, revision surgery costs, MABC neuroma treatment costs, postoperative rehabilitation, indirect productivity costs, and willingness-to-pay thresholds.

Together, Tables 1-3 define the inputs used in the PSA and Monte Carlo simulation.

## Results

The reproducible Python implementation generated the following PSA outputs. To maximize reproducibility, the reported values are generated directly from the accompanying Python implementation (Table 4).

**Table 4:** Reproducible PSA outputs by severity subgroup and arm (N=10,000 iterations) MABC: medial antebrachial cutaneous; SD: simple decompression; TMT: transmuscular anterior transposition; QALY: quality-adjusted life year; PSA: probabilistic sensitivity analysis; CrI: credible interval.

Severity	Arm	5-year QALYs, mean (95% CrI)	5-year cost, mean (95% CrI)	Lifetime MABC probability
Mild	SD	3.941 (3.880-4.003)	$6,490 ($5,444-$7,660)	2.40%
Mild	TMT	3.872 (3.809-3.932)	$9,282 ($7,762-$10,972)	7.00%
Axonal	SD	3.812 (3.742-3.883)	$15,392 ($11,262-$20,788)	4.90%
Axonal	TMT	3.817 (3.747-3.885)	$15,713 ($11,955-$20,452)	7.20%

In mild/demyelinating disease, SD generated higher QALYs and lower costs than TMT, making SD the dominant strategy in the reproducible model. This finding is consistent with current practice patterns favoring decompression for patients without subluxation or advanced axonal loss [2-4] (Table 5).

**Table 5:** Incremental analysis of TMT versus SD CE: cost-effective; ICER: incremental cost-effectiveness ratio; QALY: quality-adjusted life-year; SD: simple decompression; TMT: transmuscular anterior transposition. In the mild/demyelinating stratum, TMT yielded fewer QALYs at a higher cost and is therefore dominated, so a positive ICER is not defined. In the axonal stratum, the incremental QALY difference straddled zero across iterations, rendering the ICER ratio uninterpretable; the probability of cost-effectiveness and the dominance frequencies are reported instead. Values are means across 10,000 Monte Carlo iterations.

Severity	Delta QALY	Delta cost	Median ICER	P(TMT CE) at $50k/QALY	P(TMT CE) at $100k/QALY	TMT dominant	TMT dominated
Mild	-0.069	$2,793	Dominated	0.60%	1.80%	0.10%	93.60%
Axonal	0.005	$321	Not meaningful	50.00%	51.70%	28.80%	29.00%

In axonal disease, TMT generated a small mean QALY advantage but at higher mean cost. The credible intervals overlapped substantially, and the probability of cost-effectiveness depended on the willingness-to-pay threshold and on the modeled good-outcome differential between TMT and SD in axonal disease. This result should therefore be interpreted as equipoise with a possible advantage for TMT in selected axonal patients rather than as definitive evidence of superiority.

The MABC comparison changed across severity strata. In mild disease, SD had lower lifetime MABC exposure. In axonal disease, the difference narrowed because SD had greater downstream revision exposure, and revision surgery carries a high probability of encountering or treating MABC neuroma in the published revision series [8,15,16].

## Discussion

This analysis expresses the clinically important idea of electromyography (EMG)-severity stratification, and aims to create a reproducible Markov model. The most stable conclusion is that SD remains the preferred operation for mild-to-moderate demyelinating CuTS without subluxation. It is less costly, less invasive, and associated with lower cumulative MABC exposure in this subgroup.

The axonal subgroup is more uncertain and should be presented that way. The rationale for considering TMT in axonal disease is not that the procedure is uniformly superior. Rather, the rationale is that axonal disease changes the balance of risks: recurrent compression and revision surgery may be more consequential, and the long-term MABC advantage of SD narrows once revision exposure is modeled [16]. In this setting, TMT may function as a more definitive operation for selected patients [13], but this hypothesis requires prospective validation.

Limitations

The model relies on published aggregate data and several modeled assumptions rather than patient-level EMG-stratified comparative trial data. The axonal subgroup results are especially sensitive to the assumed good-outcome differential between TMT and SD. The utility decrement for MABC injury is estimated from neuropathic pain analogs rather than directly measured in patients with confirmed MABC neuroma. The 73% MABC neuroma figure comes from a revision population and should not be interpreted as the primary-procedure iatrogenic injury rate [8]. Generic utility instruments may underestimate meaningful hand-specific improvement after cubital tunnel surgery [16]. Finally, the transmuscular technique modeled here may not generalize to all anterior transposition techniques or all surgeon experience levels [13,17].

## Conclusions

This reproducible Markov model supports SD as the dominant strategy for mild/demyelinating CuTS without subluxation. In axonal disease, the model suggests clinical equipoise with a possible role for TMT in selected patients because revision risk and cumulative MABC exposure change the long-term tradeoff. Subluxation remains an independent indication for transposition. The analysis should be interpreted as a quantitative framework for shared decision-making and as a rationale for prospective EMG-stratified comparative research.

## Appendices

Appendix A

Model Inputs, Distribution Parameters, and Annual Transition Probabilities

This section documents the base-case values and probability distributions used in the definitive reproducible implementation of the Markov model. Probabilities and utilities use beta distributions. Costs use gamma distributions. Annual revision probabilities are derived from cumulative probabilities using p_annual = 1 - (1 - p_cumulative)^(1/4) across cycles two through five.

**Table 6:** Model input distributions

Category	Arm	Severity	Parameter	Distribution	Mean	Low	High	Alpha	Beta	Shape	Scale	Source_basis
Arm probability	SD		Primary MABC injury probability	beta	0.015	0.01	0.02	34.0408	2235.345			Published/anatomic and clinical ranges; see manuscript
Arm probability	SD		Primary complication probability	beta	0.036	0.02	0.06	11.9627	320.3345			Published/anatomic and clinical ranges; see manuscript
Arm cost	SD		Primary operative cost	gamma	3500	2800	4200			96.04	36.4431	2024 USD estimates; see manuscript
Arm cost	SD		Complication cost	gamma	900	300	1800			5.5319	162.6926	2024 USD estimates; see manuscript
Arm probability	TMT		Primary MABC injury probability	beta	0.065	0.05	0.08	67.3828	969.276			Published/anatomic and clinical ranges; see manuscript
Arm probability	TMT		Primary complication probability	beta	0.096	0.06	0.14	19.9074	187.461			Published/anatomic and clinical ranges; see manuscript
Arm cost	TMT		Primary operative cost	gamma	5650	4500	6800			92.7285	60.9305	2024 USD estimates; see manuscript
Arm cost	TMT		Complication cost	gamma	1100	400	2200			5.7387	191.6815	2024 USD estimates; see manuscript
Severity utility		mild	Symptomatic utility	beta	0.73	0.72	0.74	5526.679	2044.114			Song et al. [5] /Teunissen et al. [13] and severity adjustment
Severity cost		mild	Productivity cost	gamma	2150	1200	3200			17.7578	121.0736	Societal/productivity estimate
Severity utility		axonal	Symptomatic utility	beta	0.635	0.62	0.65	2512.235	1444.04			Song et al. [5] /Teunissen et al. [13] and severity adjustment
Severity cost		axonal	Productivity cost	gamma	5400	3200	7600			23.1448	233.3133	Societal/productivity estimate
Arm-severity probability	SD	mild	Good outcome after primary surgery	beta	0.84	0.83	0.85	4336.173	825.9377			Literature-derived/modelled; see manuscript
Arm-severity probability	SD	mild	Cumulative revision probability	beta	0.08	0.05	0.11	25.0526	288.1049			Literature-derived/modelled; see manuscript
Arm-severity probability	TMT	mild	Good outcome after primary surgery	beta	0.82	0.81	0.83	4648.745	1020.456			Literature-derived/modelled; see manuscript
Arm-severity probability	TMT	mild	Cumulative revision probability	beta	0.04	0.02	0.07	9.4011	225.6268			Literature-derived/modelled; see manuscript
Arm-severity probability	SD	axonal	Good outcome after primary surgery	beta	0.6	0.52	0.68	85.836	57.224			Literature-derived/modelled; see manuscript
Arm-severity probability	SD	axonal	Cumulative revision probability	beta	0.119	0.08	0.17	23.5487	174.3399			Literature-derived/modelled; see manuscript
Arm-severity probability	TMT	axonal	Good outcome after primary surgery	beta	0.7	0.62	0.78	87.5367	37.5157			Literature-derived/modelled; see manuscript
Arm-severity probability	TMT	axonal	Cumulative revision probability	beta	0.032	0.01	0.07	4.199	127.0203			Literature-derived/modelled; see manuscript
Common cost			Revision cubital tunnel surgery	gamma	7800	6200	9500			85.8486	90.8576	Revision surgery/neurolysis complexity estimate
Common cost			Postoperative rehabilitation	gamma	1100	800	1400			51.6482	21.2979	Standard OT course estimate
Common cost			MABC neuroma management	gamma	3000	1800	4200			24.01	124.9479	Injection series and potential operative excision estimate
Common probability			Good outcome after revision surgery	beta	0.67	0.55	0.78	42.3609	20.8643			Revision series estimate
Common probability			MABC neuroma encountered/treated at revision	beta	0.73	0.6	0.84	37.6548	13.9271			Mackinnon and Novak [9] revision series; applied only to revision pathway
Utility			Postoperative improving	beta	0.715	0.68	0.76	349.1086	139.1552			Early recovery state; intentionally conservative
Utility			Postoperative good outcome	beta	0.895	0.88	0.91	1435.14	168.3684			Brauer and Graham [4]; normalized generic utility
Utility			Postoperative partial recovery/plateau	beta	0.765	0.75	0.78	2347.355	721.083			Residual hand dysfunction analog
Utility			Failed primary/recurrent symptoms	beta	0.665	0.65	0.68	2528.736	1273.875			Persistent postoperative symptoms
Utility			MABC injury permanent state, initial	beta	0.7	0.68	0.72	1411.088	604.752			Chronic neuropathic pain/painful neuroma analog
Utility			MABC annual utility decrement	beta	0.065	0.05	0.08	67.3828	969.276			Annual decrement applied while in MABC state
Utility			Post-revision good outcome	beta	0.835	0.82	0.85	1963.369	387.9711			Revision success state
Utility			Post-revision partial/poor outcome	beta	0.7	0.68	0.72	1411.088	604.752			Poor revision outcome analog

**Table 7:** Annual transition probabilities used in the base-case model logic MABC: medial antebrachial cutaneous; SD: simple decompression; TMT: transmuscular anterior transposition.

Severity	Arm	Cycle	Transition	Probability	Source/basis
Mild	SD	1	Index procedure: primary MABC injury	0.015	Primary MABC input mean
Mild	SD	1	Index procedure: good primary outcome if no MABC	0.84	Good primary outcome input mean
Mild	SD	1	Index procedure: partial/plateau if no MABC	0.16	Complement of good primary outcome
Mild	SD	2	Partial/plateau to revision	0.0206	Annualized from cumulative p=0.080 over cycles 2-5
Mild	SD	2	Revision to MABC injury pathway	0.73	MABC at revision input mean
Mild	SD	2	Revision non-MABC to good revision outcome	0.67	Good revision input mean
Mild	SD	2	Revision non-MABC to partial/poor revision outcome	0.33	Complement of good revision outcome
Mild	SD	3	Partial/plateau to revision	0.0206	Annualized from cumulative p=0.080 over cycles 2-5
Mild	SD	3	Revision to MABC injury pathway	0.73	MABC at revision input mean
Mild	SD	3	Revision non-MABC to good revision outcome	0.67	Good revision input mean
Mild	SD	3	Revision non-MABC to partial/poor revision outcome	0.33	Complement of good revision outcome
Mild	SD	4	Partial/plateau to revision	0.0206	Annualized from cumulative p=0.080 over cycles 2-5
Mild	SD	4	Revision to MABC injury pathway	0.73	MABC at revision input mean
Mild	SD	4	Revision non-MABC to good revision outcome	0.67	Good revision input mean
Mild	SD	4	Revision non-MABC to partial/poor revision outcome	0.33	Complement of good revision outcome
Mild	SD	5	Partial/plateau to revision	0.0206	Annualized from cumulative p=0.080 over cycles 2-5
Mild	SD	5	Revision to MABC injury pathway	0.73	MABC at revision input mean
Mild	SD	5	Revision non-MABC to good revision outcome	0.67	Good revision input mean
Mild	SD	5	Revision non-MABC to partial/poor revision outcome	0.33	Complement of good revision outcome
Mild	TMT	1	Index procedure: primary MABC injury	0.065	Primary MABC input mean
Mild	TMT	1	Index procedure: good primary outcome if no MABC	0.82	Good primary outcome input mean
Mild	TMT	1	Index procedure: partial/plateau if no MABC	0.18	Complement of good primary outcome
Mild	TMT	2	Partial/plateau to revision	0.0102	Annualized from cumulative p=0.040 over cycles 2-5
Mild	TMT	2	Revision to MABC injury pathway	0.73	MABC at revision input mean
Mild	TMT	2	Revision non-MABC to good revision outcome	0.67	Good revision input mean
Mild	TMT	2	Revision non-MABC to partial/poor revision outcome	0.33	Complement of good revision outcome
Mild	TMT	3	Partial/plateau to revision	0.0102	Annualized from cumulative p=0.040 over cycles 2-5
Mild	TMT	3	Revision to MABC injury pathway	0.73	MABC at revision input mean
Mild	TMT	3	Revision non-MABC to good revision outcome	0.67	Good revision input mean
Mild	TMT	3	Revision non-MABC to partial/poor revision outcome	0.33	Complement of good revision outcome
Mild	TMT	4	Partial/plateau to revision	0.0102	Annualized from cumulative p=0.040 over cycles 2-5
Mild	TMT	4	Revision to MABC injury pathway	0.73	MABC at revision input mean
Mild	TMT	4	Revision non-MABC to good revision outcome	0.67	Good revision input mean
Mild	TMT	4	Revision non-MABC to partial/poor revision outcome	0.33	Complement of good revision outcome
Mild	TMT	5	Partial/plateau to revision	0.0102	Annualized from cumulative p=0.040 over cycles 2-5
Mild	TMT	5	Revision to MABC injury pathway	0.73	MABC at revision input mean
Mild	TMT	5	Revision non-MABC to good revision outcome	0.67	Good revision input mean
Mild	TMT	5	Revision non-MABC to partial/poor revision outcome	0.33	Complement of good revision outcome
Axonal	SD	1	Index procedure: primary MABC injury	0.015	Primary MABC input mean
Axonal	SD	1	Index procedure: good primary outcome if no MABC	0.6	Good primary outcome input mean
Axonal	SD	1	Index procedure: partial/plateau if no MABC	0.4	Complement of good primary outcome
Axonal	SD	2	Partial/plateau to revision	0.0312	Annualized from cumulative p=0.119 over cycles 2-5
Axonal	SD	2	Revision to MABC injury pathway	0.73	MABC at revision input mean
Axonal	SD	2	Revision non-MABC to good revision outcome	0.67	Good revision input mean
Axonal	SD	2	Revision non-MABC to partial/poor revision outcome	0.33	Complement of good revision outcome
Axonal	SD	3	Partial/plateau to revision	0.0312	Annualized from cumulative p=0.119 over cycles 2-5
Axonal	SD	3	Revision to MABC injury pathway	0.73	MABC at revision input mean
Axonal	SD	3	Revision non-MABC to good revision outcome	0.67	Good revision input mean
Axonal	SD	3	Revision non-MABC to partial/poor revision outcome	0.33	Complement of good revision outcome
Axonal	SD	4	Partial/plateau to revision	0.0312	Annualized from cumulative p=0.119 over cycles 2-5
Axonal	SD	4	Revision to MABC injury pathway	0.73	MABC at revision input mean
Axonal	SD	4	Revision non-MABC to good revision outcome	0.67	Good revision input mean
Axonal	SD	4	Revision non-MABC to partial/poor revision outcome	0.33	Complement of good revision outcome
Axonal	SD	5	Partial/plateau to revision	0.0312	Annualized from cumulative p=0.119 over cycles 2-5
Axonal	SD	5	Revision to MABC injury pathway	0.73	MABC at revision input mean
Axonal	SD	5	Revision non-MABC to good revision outcome	0.67	Good revision input mean
Axonal	SD	5	Revision non-MABC to partial/poor revision outcome	0.33	Complement of good revision outcome
Axonal	TMT	1	Index procedure: primary MABC injury	0.065	Primary MABC input mean
Axonal	TMT	1	Index procedure: good primary outcome if no MABC	0.7	Good primary outcome input mean
Axonal	TMT	1	Index procedure: partial/plateau if no MABC	0.3	Complement of good primary outcome
Axonal	TMT	2	Partial/plateau to revision	0.0081	Annualized from cumulative p=0.032 over cycles 2-5
Axonal	TMT	2	Revision to MABC injury pathway	0.73	MABC at revision input mean
Axonal	TMT	2	Revision non-MABC to good revision outcome	0.67	Good revision input mean
Axonal	TMT	2	Revision non-MABC to partial/poor revision outcome	0.33	Complement of good revision outcome
Axonal	TMT	3	Partial/plateau to revision	0.0081	Annualized from cumulative p=0.032 over cycles 2-5
Axonal	TMT	3	Revision to MABC injury pathway	0.73	MABC at revision input mean
Axonal	TMT	3	Revision non-MABC to good revision outcome	0.67	Good revision input mean
Axonal	TMT	3	Revision non-MABC to partial/poor revision outcome	0.33	Complement of good revision outcome
Axonal	TMT	4	Partial/plateau to revision	0.0081	Annualized from cumulative p=0.032 over cycles 2-5
Axonal	TMT	4	Revision to MABC injury pathway	0.73	MABC at revision input mean
Axonal	TMT	4	Revision non-MABC to good revision outcome	0.67	Good revision input mean
Axonal	TMT	4	Revision non-MABC to partial/poor revision outcome	0.33	Complement of good revision outcome
Axonal	TMT	5	Partial/plateau to revision	0.0081	Annualized from cumulative p=0.032 over cycles 2-5
Axonal	TMT	5	Revision to MABC injury pathway	0.73	MABC at revision input mean
Axonal	TMT	5	Revision non-MABC to good revision outcome	0.67	Good revision input mean
Axonal	TMT	5	Revision non-MABC to partial/poor revision outcome	0.33	Complement of good revision outcome

Appendix B

Output Validation for Definitive Reproducible Model

The accompanying Python script is the definitive reproducible implementation of the model used for this revised manuscript. All numerical values reported in the revised manuscript were generated directly from this script, including the raw probabilistic sensitivity analysis iterations, summary outputs by treatment arm and severity stratum, incremental TMT-versus-SD outputs, and cost-effectiveness acceptability curve points across willingness-to-pay thresholds from $0 to $200,000/QALY. Earlier spreadsheet-based versions were exploratory and were used only during model development; they are not the final analytic source for the revised manuscript.

Execution environment: Python 3; NumPy and pandas required. The default script uses 10,000 Monte Carlo iterations, random seed 42, five annual cycles, and 3% annual discounting. Running the script writes four CSV outputs: raw PSA iterations, summary outputs, incremental outputs, and CEAC points.

Tables 8, 9 provide supplementary outputs from the final reproducible Python implementation.

**Table 8:** Summary outputs generated by the definitive code MABC: medial antebrachial cutaneous; SD: simple decompression; TMT: transmuscular anterior transposition; QALY: quality-adjusted life year.

Severity	Arm	QALY mean	QALY 2.5%	QALY 97.5%	Cost mean	Cost 2.5%	Cost 97.5%	MABC mean
Axonal	SD	3.8125	3.7414	3.8828	10458.41	8325.64	12972.44	0.0492
Axonal	TMT	3.8165	3.7474	3.8854	12346.29	10071.59	15058.48	0.0717
Mild	SD	3.9413	3.8797	4.0029	6898.83	5723.94	8243.56	0.0242
Mild	TMT	3.8722	3.8112	3.932	9073.12	7599.9	10698.33	0.07

**Table 9:** Incremental TMT versus SD outputs generated by the definitive code MABC: medial antebrachial cutaneous; SD: simple decompression; TMT: transmuscular anterior transposition; QALY: quality-adjusted life year;  ICER: incremental cost-effectiveness ratio.

Severity	Delta QALY	Delta cost	Median ICER	P(TMT CE) at $50k	P(TMT CE) at $100k	TMT dominant	TMT dominated
Mild	-0.0691	2174.29	-28032.5	0.0093	0.0219	0.0011	0.929
Axonal	0.004	1887.88	4505.55	0.2947	0.3893	0.0737	0.4022

These tables present the additional model-generated results used to support the primary PSA and cost-effectiveness interpretation reported in the main text.
